# exTREEmaTIME: a method for incorporating uncertainty into divergence time estimates

**DOI:** 10.1242/bio.059181

**Published:** 2022-02-11

**Authors:** Tom Carruthers, Robert W. Scotland

**Affiliations:** 1 The Jodrell Building, Royal Botanic Gardens Kew, Richmond, London TW9 3AE, UK; 2 Department of Plant Sciences, University of Oxford, South Parks Road, Oxford OX1 3RB, UK

**Keywords:** Uncertainty, Divergence times, Assumptions

## Abstract

We present a method of divergence time estimation (exTREEmaTIME) that aims to effectively account for uncertainty in divergence time estimates. The method requires a minimal set of assumptions, and, based on these assumptions, estimates the oldest possible divergence times and youngest possible divergence times that are consistent with the assumptions. We use a series of simulations and empirical analyses to illustrate that exTREEmaTIME is effective at representing uncertainty. We then describe how exTREEmaTIME can act as a basis to determine the implications of the more stringent assumptions that are incorporated into other methods of divergence time estimation that produce more precise estimates. This is critically important given that many of the assumptions that are incorporated into these methods are highly complex, difficult to justify biologically, and as such can lead to estimates that are highly inaccurate.

This article has an associated First Person interview with the first author of the paper.

## Abstract (precis)

**Summary:** We present a method for estimating evolutionary timescales that makes minimal assumptions and accurately reflects uncertainty. This enables evolutionary researchers to test hypotheses in contexts where uncertainty is adequately accounted for, and to determine the implications of the more stringent assumptions that underpin other methods.

## INTRODUCTION

Divergence time estimation plays a central role in evolutionary research. Empiricists use and estimate divergence times to investigate the timing of evolutionary diversification within different clades (Jarvis et al., 2014; Smith and Brown, 2018; Betts et al., 2018; Ramírez-Barahona et al., 2020). Meanwhile, theoreticians and methodologists seek new insights into the ways in which molecular sequence data and the fossil record can be combined to provide reliable divergence time estimates (Magallón et al., 2013; dos Reis et al., 2014; Heath et al., 2014; Höhna et al., 2016; Brown and Smith, 2018)

An important characteristic of divergence time estimation is that the primary source of data (molecular sequences) does not actually provide any information about time (Britton, 2005). This is because only the total number of substitutions separating any pair of molecular sequences can be estimated directly, which is itself a product of the substitution rate and time since the sequences diverged (Britton, 2005). As a result, divergence time estimates are entirely sensitive to assumptions about substitution rates and node ages within a phylogeny (Carruthers and Scotland, 2021a).

Initial approaches to divergence time estimation were simplistic. They involved the use of strict clock models that assumed the same substitution rate on every branch in a phylogeny, and a limited number of node calibrations where the ages of certain nodes were assumed to be known (Langley and Fitch, 1974; Britten, 1984; Gillespie, 1989, 1991; Bromham, 2006). These assumptions were generally unrealistic, meaning that divergence time estimates were often very inaccurate.

More recently developed methods attempt to account for more complex patterns of molecular evolution and the idiosyncratic nature of the fossil record (the primary source of evidence for making assumptions about node ages in a phylogeny). A range of different relaxed clock methods that incorporate among-branch-substitution-rate-variation are now widely used. These methods vary in whether substitution rates are inherited between ancestral and descendant branches (Sanderson, 1997, 2002; Thorne et al., 1998; Kishino et al., 2001; Tamura et al., 2018), whether substitution rates vary gradually (Drummond et al., 2006) or in discreet jumps in different parts of the phylogeny (Drummond and Suchard, 2011), and whether substitution rates are correlated with other traits (Lartillot and Poujol, 2011; Ho, 2014; Berv and Field, 2018). For temporal calibrations, different probability distributions are now widely used that aim to describe uncertainty in the relationship between a fossil age and the age of a calibrated node (Ho and Philips, 2009). Tip calibration, where a phylogeny is calibrated based on an explicit phylogenetic hypothesis of the relationship between fossils and extant taxa, has also recently been advocated (Heath et al., 2014). Aside from combining fossils with molecular phylogenies, modern Bayesian methods make further assumptions about time by assuming that branching events are underpinned by constant speciation and extinction rates (Drummond et al., 2006; Höhna et al., 2016).

Analyses based on these more recent methods are highly complex, and result in a wide range of interlinked assumptions being incorporated into a single analysis. Interactions among these assumptions can have complex effects on parameter estimates (Brown and Smith, 2018; Carruthers and Scotland, 2021a). Further, the interlinked assumptions are themselves hard to justify biologically. Even explicit model comparison methods have a limited utility in this context, given the computational burden of their implementation and the fact that the parameters that are estimated in divergence time estimation (substitution rates and branch time durations) are confounded (Carruthers and Scotland, 2021b).

Extreme sensitivity to a series of assumptions that are difficult to justify poses a potentially existential problem for divergence time estimation, and this problem is accentuated further by the complexity of current methods. The outcome of this is that divergence time estimates are often inaccurate, and the specific nature of many of the assumptions that are incorporated into analyses (for example, assuming that among-branch-substitution-rate-variation conforms to a specific model) means that the degree of uncertainty underlying divergence time estimates is often greatly underestimated (Carruthers and Scotland, 2021b).

Here, we present a method of divergence time estimation that is designed to accurately estimate uncertainty in divergence time estimates. It is based on a minimal set of justifiable assumptions, namely the minimum and maximum conceivable substitution rate, and potentially minimum and/or maximum age constraints for one or several nodes. The method does not use an explicit model of among-branch-substitution-rate-variation, use probability distributions for describing the relationship between fossil ages and clade ages, or make any assumptions about the speciation rate and extinction rate underpinning the phylogeny. Given that the potential range of substitution rates and minimum and maximum age for any particular node are very poorly known for many groups, the method is likely to lead to very uncertain divergence time estimates. Further, given that the purpose of the method is to incorporate uncertainty accurately into divergence time estimates, it exclusively estimates the minimum and maximum divergence time estimates that are consistent with a set of assumptions, rather than a mean or most probable age for a particular clade.

**Fig. 1.**
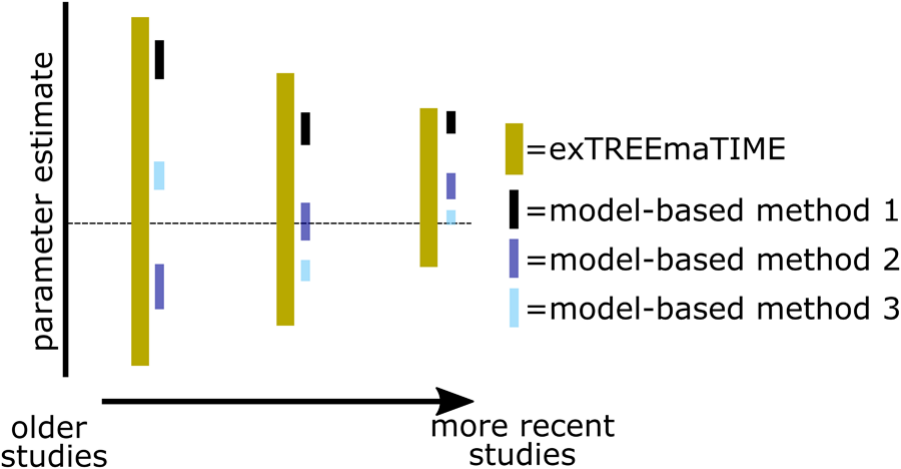
**An illustration of the rationale for exTREEmaTIME.** The horizontal dashed line is the correct value. Each group of four error bars represents parameter estimates from a series of sequentially recent studies in which knowledge of substitution rates and the fossil record has improved. Analyses from exTREEmaTIME always incorporate the correct value. Meanwhile, analyses with model-based methods underrepresent uncertainty in divergence time estimates such that they do not always incorporate the correct value. Further, more recent studies can directly contradict older studies in cases where the error bar from the more recent study does not overlap with that of the older study.

The power of this method lies in its ability to represent the full extent of uncertainty in divergence time estimates in a single analysis (Fig. 1). Once this has been quantified, methods incorporating more specific assumptions can be implemented (Fig. 1). By performing these subsequent analyses in the context of knowledge of the uncertainty underlying divergence time estimates, it is possible to make more robust statements about the implications of assumptions underlying these methods, and compare the probability of the biological conclusions that are implied by the results from each method. Such a comparative framework is useful for methodologists aiming to understand the implications of different assumptions, and empiricists who aim to understand the implications of assumptions for biological conclusions about a specific group. Below, we perform a series of simulations and empirical analyses to illustrate this rationale.

## RESULTS

### Simulation experiments

**Fig. 2.**
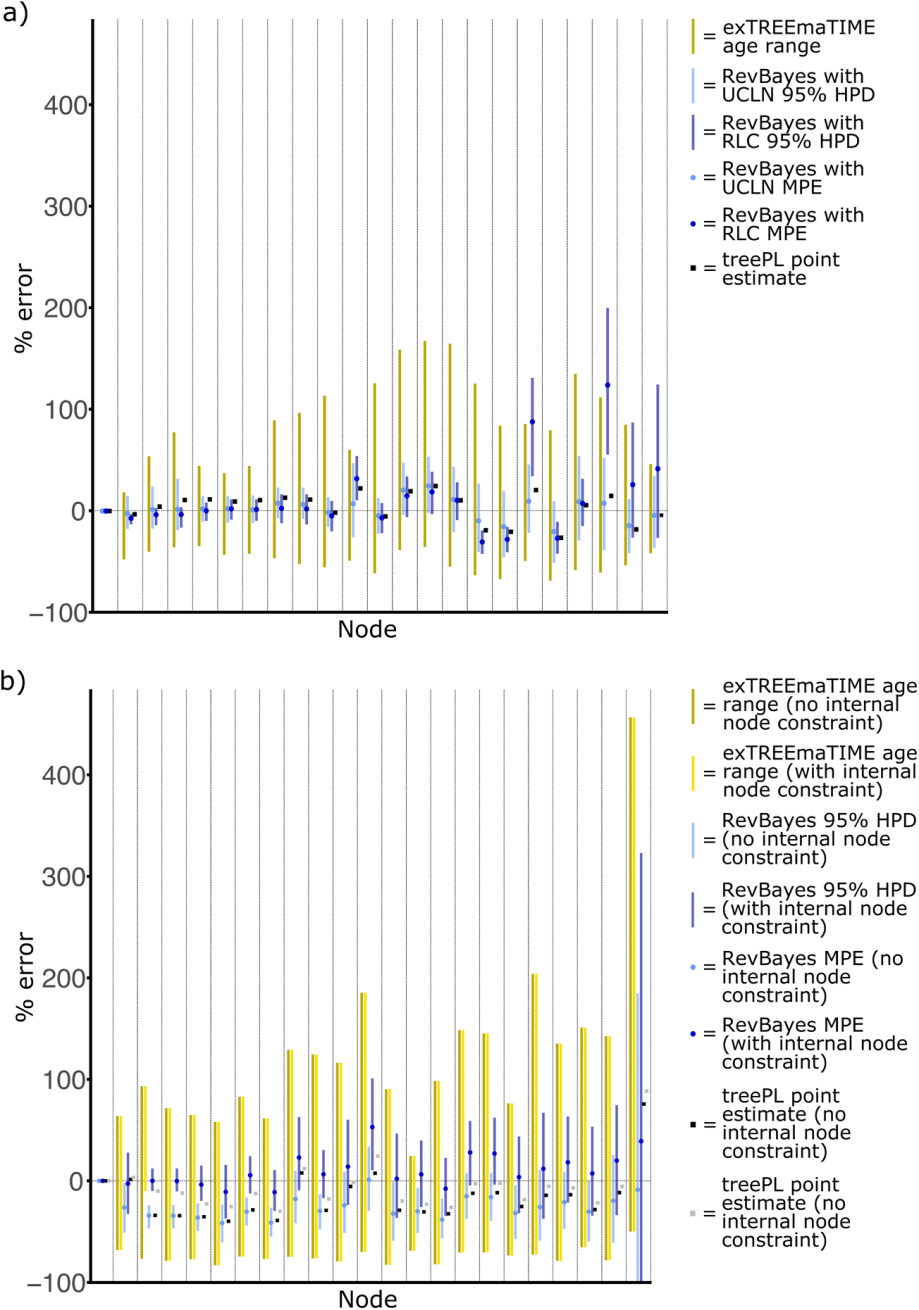
**Results from simulation experiments summarising error in estimated node ages.** (A) is when the simulated evolutionary process has a constant diversification rate. (B) is when the simulated evolutionary process has an increase in the speciation rate. *MPE, mean posterior estimate; HPD, highest posterior density.

In the first simulation, the data was simulated along a branching process in which the speciation rate was constant, and the substitution rate for each branch corresponded to an uncorrelated lognormal relaxed clock (UCLN). Divergence times were then estimated in exTREEmaTIME, RevBayes (Höhna et al., 2016), and treePL (Smith and O'Meara, 2012). In RevBayes, analyses were performed with a UCLN (corresponding to the model under which the data was simulated) or a random-local-clock (RLC). Violation of the substitution rate model could then be tested, with estimates compared to those obtained with exTREEmaTIME. Overall, analyses performed in exTREEmaTIME produced the widest ranges for feasible age estimates (Fig. 2A), and the range always incorporated the correct value. For RevBayes and a UCLN relaxed clock, estimates were more precise, and always incorporated the correct value (Fig. 2A). By contrast, with an RLC the correct value was often not incorporated in the 95% HPD (Fig. 2A). Analyses in treePL varied about the mean somewhat, although they did not depart significantly, and the divergence time estimate from treePL was always incorporated in the 95% HPD that was estimated with RevBayes and a UCLN (Fig. 2A).

A subsequent simulation differed in that the branching process from which the data was simulated had an increase in the speciation rate for a clade, thus violating the assumption of constant speciation and extinction rates that underpins most methods of Bayesian divergence time estimation. The simulated data was then analysed in two settings. First, analyses were performed with no node calibrations for internal nodes, and subsequently, analyses were performed where the crown node for the clade with an increase in the speciation rate had a minimum constraint that was 10% below the correct value. The utility of a node calibration is therefore assessed in a more complex example, where the distribution of clade ages does not correspond to a simple branching process with constant rates. In this simulation, analyses using exTREEmaTIME always included the correct value within the range of feasible age estimates (Fig. 2B). The implementation of the node calibration increased the minimum age estimate for the specific node with the node calibration, but had no impact on other nodes throughout the rest of the tree (Fig. 2B). For analyses in RevBayes, and without the node calibration, the 95% HPD did not incorporate the correct value for a high proportion of nodes (Fig. 2B). This situation was rectified partially when the node calibration was implemented, although some biases were induced at other nodes (Fig. 2B). For analyses in treePL, estimates deviated considerably from the correct value. They did nonetheless overlap with the 95% HPDs from RevBayes, and the implementation of the node calibration tended to reduce the degree of error (Fig. 2B).

### Empirical analyses

**Fig. 3.**
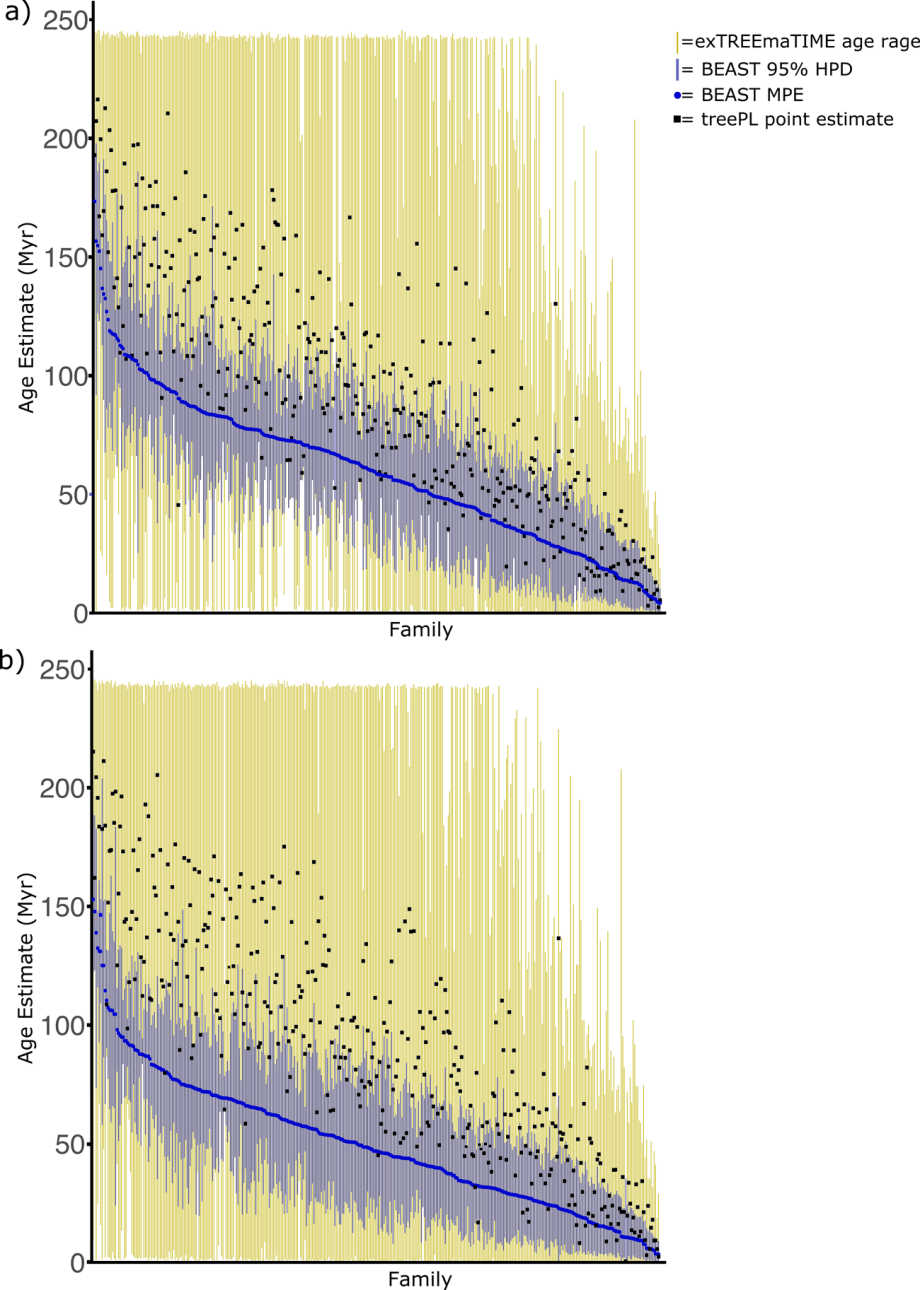
**Comparison of angiosperm family crown age estimates.** In A the full dataset of fossil calibrations from Ramírez-Barahona et al. (2020) is used, in B a subset of fossil calibrations that are assigned to nodes based on formal phylogenetic analyses is used.

Crown age estimates for angiosperm families estimated in BEAST and treePL by Ramírez-Barahona et al. (2020) were compared to those estimated in exTREEmaTIME using the same dataset. Age estimates with exTREEmaTIME were far less precise than those estimated in BEAST when either the full set of fossil calibrations were used (Fig. 3A), or when the subset of 45 fossils selected by Ramírez-Barahona et al. (2020) that had undergone formal phylogenetic analyses were used (Fig. 3B). However, the full dataset of fossil calibrations led to an increase in the minimum age estimate for many families, especially with exTREEmaTIME (Fig. 3). Note that divergence time estimates from treePL always differed markedly from those obtained with BEAST such that the divergence time estimate from treePL often lay outside the 95% HPD from BEAST. By contrast, age estimates from treePL lie within the range of feasible ages estimated by exTREEmaTIME (Fig. 3).

## DISCUSSION

### exTREEmaTIME produces imprecise divergence time estimates that account for uncertainty

Age estimates from exTREEmaTIME were far less precise than those estimated in a Bayesian framework or with treePL (Figs 2 and 3). This was the case in the simulation experiments and empirical analyses, and occurred even though the same molecular sequence datasets were analysed and the same node calibrations were used. This result occurs because exTREEmaTIME makes less stringent assumptions, and divergence time estimates are highly sensitive to assumptions, given that time is not estimated directly from the molecular sequence data.

The importance of the assumptions implemented in treePL and Bayesian methods is further highlighted by the fact that estimates from these methods often do not overlap (i.e., point estimates from treePL lie outside the 95% HPD from Bayesian analyses) (Figs 2 and 3), and by the fact that biologically realistic violations of assumptions lead to erroneous divergence time estimates. For example, in the simulations, the implementation of the wrong molecular clock model, or a speciation rate shift in the simulated evolutionary process (which violates the assumption of a branching process with constant rates that is used in RevBayes), causes erroneous divergence time estimates (Fig. 2). By contrast, given that exTREEmaTIME is not model based, its estimates are highly imprecise, but generally overlap with those of other methods, and always incorporate the correct value in the simulation experiments (Figs 2 and 3). exTREEmaTIME is therefore effective at accounting for uncertainty.

### A useful interpretation of divergence time estimates from exTREEmaTIME

The imprecise divergence time estimates from exTREEmaTIME can be challenging to interpret. Specifically, we suggest that exTREEmaTIME should be interpreted as a baseline that expresses the maximum level of uncertainty surrounding clade age estimates. In this vein, we are not disputing the findings of Ramírez-Barahona et al. (2020), or necessarily suggesting that exTREEmaTIME should be used as a basis to dispute any other finding. Instead, we are using exTREEmaTIME to highlight that more precise estimates are a direct result of more precise methodological assumptions. It is the job of authors of studies such as Ramírez-Barahona et al. (2020) to provide a biological justification for the assumptions that they make.

A recent and valid trend in divergence time analyses is to implement a range of methods in the hope that they arrive at some level of consistent conclusion (Morris et al., 2018; Fernández-Mazuecos et al., 2020). The rationale for this approach is that although each method is undoubtedly limited, achieving consistency with different methods must imply that some level of coherent temporal signal is being extracted from the data. Unfortunately, such comparisons are necessarily limited in scope, with often only a limited number of methods being compared. As such, the range of assumptions that are actually explored is often relatively narrow. The coherent temporal signal which is believed to have been obtained therefore reflects the limited nature of the assumptions that are explored. Even in studies where age estimates differ markedly between methods (Barba-Montoya et al., 2018; Ramírez-Barahona et al., 2020), and researchers attempt to use this as a basis to bracket uncertainty in the age estimate for a particular node, the age bracket simply represents the assumptions of the different methods that are explored.

In light of this, a comparison of different methods in the context of an exTREEmaTIME analysis can illustrate the extent to which outputs from these different methods reflect the maximum conceivable level of uncertainty. Parts of the potential age range for a clade that are estimated by exTREEmaTIME, but were not evident in previous analyses, can therefore be interrogated, and researchers can determine whether these parts of the age range are feasible. In some cases, researchers will have justifiable reasons to exclude parts of the age range expressed by exTREEmaTIME. Alternatively, following analysis with what is considered a biologically justifiable set of models, the exTREEmaTIME analysis may be refined such that it produces more precise estimates that better reflect the true level of uncertainty. Regardless, the purpose of exTREEmaTIME is to represent the maximal level of uncertainty that can be associated with divergence time estimates, and shifts the burden on those implementing other methods that result in higher levels of precision to provide biological justification for these methods.

Therefore, in presenting this method we are not seeking to undermine the search for increasingly precise divergence time estimates, because precise divergence time estimates can be an extremely powerful tool for the study of evolution. Instead, the search for precision is not wholly incompatible with the very different method being presented here that focuses on uncertainty. In our view, when these two approaches are combined, they can help researchers to better understand and embrace the uncertainty that underpins divergence time analyses.

## MATERIALS AND METHODS

### Overview of exTREEmaTIME

The method requires three types of input: a phylogeny with molecular branch lengths that reflect the number of substitutions; a maximum and minimum conceivable substitution rate across the input phylogeny (*r_max_* and *r_min_*); and minimum and maximum age constraints for nodes in the phylogeny.

Based on these inputs, two time-calibrated phylogenies are estimated, *Tree_max_* and *Tree_min_*. Respectively, these represent the maximum and minimum divergence time estimates that are consistent with the inputs. The method does not attempt to estimate a ‘most likely’ divergence time between the ages represented in *Tree_min_* and *Tree_max_*. This is because it does not make any assumptions about the nature of variation in *r* or the relationship between fossil ages and clade ages (other than the simplistic assumptions specified in the inputs), and its purpose is simply to generate feasible divergence time ranges that are consistent with the inputs.

*r_min_* and *r_max_* can be specified in two different ways. In some cases, it may be feasible to consult the relevant literature (e.g. Yang et al., 2017), although substitution rate estimates from the literature are often themselves based on important assumptions. Alternatively, exTREEmaTIME includes a function (SetAutoRates) for specifying *r_min_* and *r_max_* based on the molecular branch length variance of terminal branches in the input tree. Nonetheless, specifying *r_min_* and *r_max_* is challenging (for example branch length variation for terminal branches may not be representative of substitution rate variation across the entire tree) and there is an element of subjectivity, just as there is with specifying clock models for all other methods of divergence time estimation. Unlike other methods however, specified input parameters are not designed to encapsulate the underlying evolutionary process, but are instead designed to provide a basis for representing a maximum feasible age range for a clade. This places less of a burden on the assumptions that are implemented in the analysis. In light of this characteristic of exTREEmaTIME, we advise users to always favour wider intervals between *r_max_* and *r_min_* unless there are explicit reasons not to do so. Nonetheless, given the simplicity of the assumptions that are implemented, it is straightforward to assess the implications of different specifications for *r_min_* and *r_max_*.

exTREEmaTIME is freely available at: https://github.com/TomCarr/exTREEmaTIME. There are also extensive instructions in this repository.

### Simulation experiments

Simulations were undertaken to provide a simple context for evaluating the performance of exTREEmaTIME relative to other methods, when either the assumptions of the other methods were consistent with the simulated data, or when there were basic violations of the assumptions. Specifically, we compared the performance of exTREEmaTIME to RevBayes, a Bayesian framework that implements commonly used models in divergence time estimation, and treePL, a widely used penalised-likelihood method which assumes that substitution rates are autocorrelated.

#### Generating simulated datasets

Simulations were based on two different 24-taxon branching processes generated with a custom R script that required the package TreeSim (Stadler, 2019). For both branching processes the extinction rate was 0. In one branching process the speciation rate was constant, while in the other there was an approximately 10-fold increase for a clade. The branch lengths of these branching processes were initially in units of time*.*

Branch lengths were transformed to molecular branch lengths (reflecting the number of substitutions) by dividing them by the substitution rate. The substitution rate for each branch was drawn from a lognormal distribution (*m*=0.05 and *v*=0.00025). Molecular sequences of 50,000 bp were then simulated along these transformed branch lengths according to a JC model.

#### Analysis of simulated data

Phylogenies with molecular branch lengths were first estimated from the simulated sequence data. This step was performed in RevBayes, with the analysis constrained to the correct topology.

For subsequent divergence time estimation in exTREEmaTIME, SetAutoRates was used to define plausible values for *r_max_* and *r_min_*, with the mean posterior molecular branch lengths estimated in RevBayes used as the input. The root age was assumed to be known.

For divergence time estimation in RevBayes, two different molecular clock models were used: an uncorrelated lognormal relaxed clock (UCLN), and a random local clock (RLC). The prior distributions for these relaxed clocks were derived from the posterior distributions of molecular branch lengths estimated in RevBayes (described above). For UCLN, *m* was calculated by dividing the mean root to tip distance in the input tree (the tree estimated in RevBayes, described above) by the root age, with the correct root age assumed to be known. *v* was calculated from the variance of all terminal pairs of sister branches in the input tree. For RLC, values by which the substitution rate is multiplied were drawn from a uniform distribution, with the range of the distribution being defined by the largest branch length difference between terminal pairs of sister branches in the input tree. The probability of a change in the substitution rate on a given branch was drawn from an exponential distribution with rate=10. The substitution rate for the basal two branches of the tree was parameterised in the same manner as with UCLN. In all cases a JC model of sequence evolution was used, a Yule model was used as the branching process, and the root age was assumed to be known.

For divergence time analyses in treePL, the mean posterior molecular branch lengths estimated in RevBayes were used as input. Cross-validation was used to define the optimal smoothing parameter, with the root age assumed to be known.

For analyses in exTREEmaTIME, RevBayes, and treePL, and where sequence data had been simulated on a tree with a shift in diversification rates, a single minimum age constraint was used at the crown node of the clade for which there was an increase in the net diversification rate. This minimum age constraint was set to an age of 10% less than the true age of the clade. For analyses in RevBayes, this age constraint was implemented using a uniform distribution, where the minimum of the uniform distribution was equal to the minimum age constraint, and the maximum of the uniform distribution was equal to maximum age constraint at the root node (i.e. the maximum possible age for any node in the phylogeny).

### Empirical analyses

Empirical analyses were undertaken to evaluate the performance of exTREEmaTIME relative to other methods in the context of a large and complex biological dataset. Family crown age estimates from Ramírez-Barahona et al. (2020) were compared to those estimated in exTREEmaTIME. The comparison was based on the unconstrained calibration strategy from Ramírez-Barahona et al. (2020) in which the crown node of angiosperms had a maximum age constraint of 247Ma. To estimate divergence times with this dataset in exTREEmaTIME, we used the phylogeny estimated in RAxML by these authors as the input tree. SetAutoRates was used to estimate *r_min_* and *r_max_*. Analyses were performed with the full dataset of fossil calibrations used by Ramírez-Barahona et al. (2020), or the reduced dataset comprising fossils that could be assigned to clades based on formal phylogenetic analyses.
